# Gypenoside-Induced Apoptosis via the PI3K/AKT/mTOR Signaling Pathway in Bladder Cancer

**DOI:** 10.1155/2022/9304552

**Published:** 2022-03-29

**Authors:** Xiuming Li, Hui Liu, Chengcheng Lv, Jun Du, Fangchao Lian, Shouyi Zhang, Zhiyong Wang, Yu Zeng

**Affiliations:** ^1^Department of Urology, Affiliated Hospital of Chengde Medical University, Chengde, Hebei 067000, China; ^2^Department of Pharmacology, College of Traditional Chinese Medicine, Chengde Medical University, Chengde, Hebei 067000, China; ^3^Department of Urology the Cancer Hospital of China Medical University & Liaoning Cancer Hospital and Institute, Shenyang, Liaoning 110042, China; ^4^Tianjin Medical University Cancer Institute and Hospital, National Clinical Research Center for Cancer, Tianjin 300060, China; ^5^Key Laboratory of Cancer Prevention and Therapy, Tianjin 300060, China; ^6^Tianjin's Clinical Research Center for Cancer, Tianjin 300060, China

## Abstract

*Gynostemma pentaphyllum* (Thunb.) Makino (*G. pentaphyllum*) is a natural herbal drug that has been widely used to treat many diseases. The antitumor effects of *G. pentaphyllum* were first described in the illustrated catalog of plants. Gypenosides are the major active components of *G. pentaphyllum*, and they have been widely reported to possess antitumor effects in prostate cancer, gastric cancer, hepatocellular carcinoma, colon cancer, lung cancer, and breast cancer. However, research on the use of gypenoside in the treatment of bladder cancer has not been conducted. In this study, we explored the potential molecular mechanisms of gypenosides in the treatment of bladder cancer using network pharmacology and experimental validation. First, we used a network pharmacology–based method to identify both the effective components of gypenosides and the molecular mechanism underlying their antibladder cancer effects. The results were further confirmed by molecular docking, CCK8 and colony formation assays, and cell cycle and cell apoptosis analyses. Additionally, a mouse xenograft model of bladder cancer was used to investigate the antitumor effect of gypenosides in vivo. We identified 10 bioactive ingredients and 163 gene targets of gypenosides. Network exploration suggested that VEGFA, STAT3, and PI3KCA may be candidate agents for the antibladder cancer effect of gypenosides. In addition, analysis of the Kyoto Encyclopedia of Genes and Genomes pathway revealed that the phosphatidylinositol-3-kinase (PI3K)/AKT/mammalian target of rapamycin (mTOR) signaling pathway may play a crucial role in the mechanism of action of gypenosides against bladder cancer. Molecular docking revealed that gypenosides combine well with PI3K, AKT, and mTOR. As expected, gypenosides displayed apoptosis-inducing properties in bladder cancer cells by inactivating the PI3K/AKT/mTOR signaling pathway in vitro. Furthermore, gypenosides significantly (*P* < 0.05) inhibited the growth of bladder cancer cells in vivo. Mechanistically, gypenosides induced the apoptosis of bladder cancer cells via inactivation of the PI3K/AKT/mTOR signaling pathway.

## 1. Introduction

Bladder cancer is one of the most diagnosed urological cancers worldwide, and its incidence is particularly high in developed countries, as well as certain countries in Northern Africa and Western Asia [1]. In the United States, approximately 81,400 cases of bladder cancer were predicted to be diagnosed in 2020, and 17980 patients died of the disease [2]. Smoking and occupational toxins increase the risk of bladder cancer [3]. Non-muscle-invasive bladder cancers are treated with endoscopic resection and adjuvant intravesical therapy, while patients with muscle-invasive diseases are generally treated with radical cystectomy and urinary diversion [4]. Bladder cancer is lethal once metastases occur [5], although platin-based chemotherapy and immune checkpoint inhibitors have led to increased survival in some patients [6, 7]. This study is interested in the application of traditional Chinese medicine in antibladder cancer research.

*G. pentaphyllum* is a perennial plant of the Cucurbitaceae family that is widely distributed in China, Japan, and South Korea [8]. *G. pentaphyllum* contains saponins, flavonoids, polysaccharides, and other chemical components [9]. In traditional medicine, *G. pentaphyllum* has been used to treat diabetes, dyslipidemia, and inflammation [10]. In addition, *G. pentaphyllum* is widely used in drinks, face washes, and bath oils due to its health benefits [11]. Modern medical research has shown that *G. pentaphyllum* exhibits potent anticancer activities in hepatocellular carcinoma [12], colorectal cancer [13], and lung cancer [14].

Gypenosides are the major active components of *G. pentaphyllum* and have widespread pharmacological actions, including antihepatitis [15], antiaging [16], antihyperglycemia [17], anti-inflammatory [18, 19], immunomodulatory, and neuroprotective effects [20, 21]. Previous studies have shown that gypenosides regulate multiple cancer pathways, including DNA damage repair inhibition, induction of apoptosis, and cell cycle arrest [11]. Recently, several studies have also reported the antitumor effects of gypenosides in a variety of cancers, including hepatocellular carcinoma [22, 23], oral cancer [24, 25], lung cancer [26], prostate cancer [27], glioma tumor [28], and colorectal cancer [29]. Although the antitumor role of gypenosides has been described, whether and how gypenosides function in the treatment of bladder cancer remains elusive.

**Figure 1:**
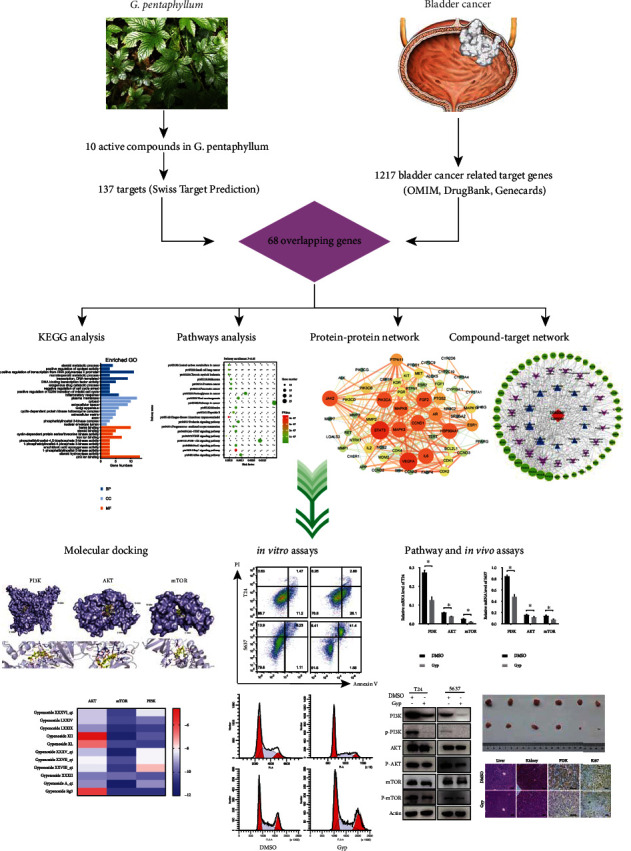
Flow chart of this study.

To evaluate the therapeutic potential of gypenosides in bladder cancer, we applied a network pharmacology approach to identify the signal pathways that are both affected by gypenosides and potentially take part in the development of bladder cancer. We further verified the network pharmacology analysis results using molecular docking and in vitro and in vivo experimental approaches. A flowchart of this study is shown in Figure 1.

## 2. Materials and Methods

### 2.1. Identification of the Potential Molecular Targets of Gypenosides

The Traditional Chinese Medicine Systems Pharmacology Database and Analysis Platform (http://lsp.nwu.edu.cn/tcmsp.php) was used to identify the active components of gypenosides and results were mainly obtained based on two ADME (Absorption, Distribution, Metabolism, and Excretion) attribute values: oral bioavailability (OB) ≥ 30% and drug similarity (DL) ≥ 0.18. We obtained the molecular structure of the compounds from the PubChem (https://pubchem.ncbi.nlm.nih.gov/) database. The ChemDraw software (version 18.0; PerkinElmer, USA) was used to describe the molecular structure of gypenosides. The SwissTargetPrediction (http://swisstargetprediction.ch/) was used to predict the potential molecular targets of gypenosides.

### 2.2. Identification of Bladder Cancer-Related Genes

The bladder cancer-associated human genes were comprehensively retrieved from three databases: DrugBank (https://go.drugbank.com/), GeneCards (https://www.genecards.org/), and OMIM (https://www.omim.org/) by searching for the keyword “bladder cancer.” Duplicate values were removed by comparing the results of the three databases.

### 2.3. Protein–Protein Interaction (PPI) Networks

The Venny 2.1.0 (http://bioinfogp.cnb.csic.es/tools/venny/index.html) was used to determine the relationship between gypenoside targets and the genes associated with bladder cancer. Then, a PPI network model was established by submitting the intersecting targets to the STRING 11.0 database (https://string-db.org). To make the results more reliable, the minimum necessary interaction score was set to “high confidence” (>0.7). All networks were visualized utilizing the Cytoscape 3.7.1 software.

### 2.4. Pathway Enrichment Analysis

Common targets were uploaded to DAVID (https://david.ncifcrf.gov/summary.jsp), using the Kyoto Encyclopedia of Genes and Genomes (KEGG) pathway analysis and gene ontology (GO) enrichment analysis. Cellular component (CC), molecular function (MF), and biological processes (BP) in GO were selected to annotate gene function. KEGG pathway annotation results confirmed the important regulatory pathways of gypenosides in bladder cancer.

### 2.5. Molecular Docking

The three-dimensional structures of the three targets we used were accessed from the Protein Databank (https://www.rcsb.org/). All the water molecules and the binding substances were removed using PyMOL. The AutoDock Tools 1.5.6 software was used to add all the hydrogens, calculate Gasteiger charges for the structure, and save them as receptors in the PDBQT file format. The structures of the gypenosides were optimized using the MM2 force field and saved in the PDBQT format as docking ligands. The grid center for molecular docking was determined using the cocrystallized ligand of the target protein complex. The AutoDock Vina 1.1.2 was used for docking, and the spacing and exhaustiveness were set to 0.375 and 8, respectively. A Lamarckian genetic algorithm was used for conformational searches. Further constraints in AutoDock Vina were set to default unless otherwise noted. The Discovery Studio 2019 was used to visualize the optimal binding affinity of the compounds. When the binding energy was < –7 kcal/mol, and we assumed a strong binding affinity between the targets and the gypenosides.

### 2.6. Reagents

*G. pentaphyllum* (GP2016-01) was collected from Zhangzhou (Fujian, China) and stored at 4°C at the Key Laboratory of Ethnomedicine of the Ministry of Education, Minzu University of China. We extracted gypenosides from *G. pentaphyllum* using the method previously described by Liu et al. [30]; the purity of the gypenosides in our study was greater than 98%. The gypenosides were stored at 4°C and dissolved in dimethyl sulfoxide (DMSO; 500-mg/ml stock solution).

### 2.7. Cell Lines and Culture

The Chinese Academy of Sciences Committee (Beijing, China) provided the T24 and 5637 bladder cancer cell lines. All human bladder cancer cells were cultured in RPMI-1640 medium supplemented with 10% fetal bovine serum (Thermo Fisher Scientific) and penicillin-streptomycin (Gibco) at 37°C in a 5% CO_2_ incubator.

### 2.8. Cell Proliferation Assay

Bladder cancer cells were counted and plated in 96-well plastic dishes (8000 cells/well) 24 h before gypenoside treatment. The selected wells were cultured in a medium supplemented with various concentrations of gypenosides (0, 200, 400, 600, 800, 1000, and 1200 *μ*g/mL). The control groups were cultured in medium alone. After 24 h of incubation, 10 *μ*L CCK8 (DOJINDO) solution was added to each well, and the plates were incubated at 37°C for 1.5 h. The optical density at 450 nm was measured using the FLUOstar Omega system (BMG Labtech GmbH, Germany), and the IC_50_ values for each cell line were calculated.

### 2.9. Colony Formation Assay

Bladder cancer cells were seeded in 12-well plates at a density of 500 cells/well. After 10–14 days of incubation, cell colonies were fixed. The T24 and 5637 cells were treated with 550 *μ*g/mL and 180 *μ*g/mL gypenosides (IC_50_ values), with the doses determined by a cell proliferation assay. The cell colonies were stained with 0.5% crystal violet, we performed three biological replicates and counted colonies using the ImageJ software (NIH USA).

### 2.10. Apoptosis and Cell Cycle Assays

Bladder cancer cells were treated with or without gypenosides for 24 h and harvested to determine the effect of gypenosides on apoptosis and the cell cycle. Cells were washed twice with cold PBS and then 100 *μ*L of solution (10^5^ cells) was transferred to a 5 ml culture tube. Next, 5 *μ*L Annexin V–FITC (BD Biosciences) and 5 *μ*L propidium iodide (PI) (BD Biosciences) were added to the tube. The solution was then gently vortexed and incubated for 15 min at room temperature in the dark. Next, 400 *μ*L of 1× binding buffer was added to each tube. A FACScan flow cytometer (BD Biosciences) was used to detect stained cells, and the data were analyzed using the FlowJo V10 software (FlowJo, USA). To identify the cell cycle phase, cells were washed twice with cold PBS and incubated with 70% ethanol at 4°C for 12 h. The cells were stained with PI (BD Biosciences) and tested within 24 h. Flow cytometry (BD Biosciences) was used to explore the cell cycle distribution.

### 2.11. RT-qPCR

Total RNA was extracted using a Total RNA Isolation Kit (RC101-01, Vazyme) in accordance with the manufacturer's protocol. Total RNA was quantified using the NanoDrop 2000 (Thermo Fisher Scientific). Total RNA was reverse transcribed into cDNA using the HiScript® III All-in-one RT SuperMix (R333-01, Vazyme). The ChamQ SYBR Color qPCR Master Mix (Q411-02, Vazyme) was used for two-step real-time RT-PCR analysis. The following primer sequences were used: PIK3CA (forward: 5′-AGTAGGCAACCGTGAAGAAAAG-3′, reverse: 5′-GAGGTGAATTGAGGTCCCTAAGA-3′). AKT (forward: 5′- GTCATCGAACGCACCTTCCAT-3′, reverse: 5′-AGCTTCAGGTACTCAAACTCGT-3′). mTOR (forward: 5′- GCAGATTTGCCAACTATCTTCGG-3′, reverse: 5′- CAGCGGTAAAAGTGTCCCCTG-3′). GAPDH (forward: 5′- GGAGCGAGATCCCTCCAAAAT-3′, reverse: 5′- GGCTGTTGTCATACTTCTCATGG-3′). The relative target gene expression levels were calculated using the *Δ*CT method.

### 2.12. Western Blotting

Bladder cancer cells were lysed using a radioimmunoprecipitation assay (RIPA) buffer (P0013K; Beyotime Biotechnology). A BCA protein analysis kit (P0010; Beyotime Biotechnology) was used to detect protein concentrations. Protein denaturation was performed at 99°C for 5 min. Protein samples were loaded into 10% SDS-PAGE, which was followed by isolation by electrophoresis and transfer to 0.45 *μ*m PVDF membranes (Millipore, USA). After that, membranes were blocked in 5% skimmed milk at room temperature for 1 h, then the following primary antibodies were added and incubated overnight at 4°C: antiphosphoinositide 3-kinase (PI3K) (EM1701-62, HUABIO, 1: 500), anti-p-PI3K (Y607) (AP1280, ABclonal, 1: 500), anti-AKT (4691S, Cell Signaling Technology, 1: 1000), anti-p-AKT (Ser473) (4060S, Cell Signaling Technology, 1: 2000), anti-mTOR (2983S, Cell Signaling Technology, 1: 1000), anti-p-mTOR (Ser2448) (2971S, Cell Signaling Technology, 1: 1000), anti-Bcl2 (15071S, Cell Signaling Technology, 1: 1000), anti-Bax (2772S, Cell Signaling Technology, 1: 1000), anti-caspase-9 (ab32539, Abcam, 1: 1000), anti-CDK2 (sc-6248, SANTA, 1: 1000), anti-CDK4 (sc-23896, SANTA, 1: 1000), and anti-Cyclin D1 (sc-8396, SANTA, 1: 1000). Subsequently, the membranes were incubated with the secondary antibody at room temperature for 1 h. The membranes were washed three times with TBST for 5 min each, and immunoblotting was performed using enhanced chemiluminescence (Thermo Fisher Scientific).

### 2.13. Xenograft Tumor Model

Liaoning Changsheng Biotechnology Co., Ltd. (Benxi, China) provided BALB/c male nude mice aged 4–6 weeks (14–16 g). The animals were kept in a pathogen-free environment for all experiments. Following the recommendations of the China Medical University Ethics Committee (CMU2021375) and the Declaration of Helsinki, xenograft tumor models were established in nude mice. We randomly divided the 12 mice into two groups based on weight, with each group containing six mice. Equivalent volumes (1 × 10^7^) of 5637 cells were implanted bilaterally into the flanks of the mice. When the tumors could be palpated and detected (3–4 weeks), mice were randomly assigned to different treatment groups. Gypenosides (100 mg/kg) were orally administered every day, and saline solution was used as a control. The weight and tumor diameters of the mice were measured weekly. After 35 days of treatment, the mice were euthanized, and tumor specimens were collected, photographed, measured, and immunohistochemically examined.

### 2.14. Immunohistochemistry

We prepared the samples from the xenograft tumor mice using formalin-fixed, paraffin-embedded samples. Paraffin-embedded samples were cut into 4 *μ*m thick sections, which were then blocked with 3% hydrogen peroxide for 60 min at room temperature. After antigen retrieval, the sections were incubated with antibodies against PI3K (EM1701-62, HUABIO, 1: 200) and Ki-67 (9449 s, Cell Signaling Technology, 1: 500). After incubation with the primary antibodies, the tissue sections were incubated with the appropriate secondary antibodies (BM3895, BOSTER Biological Technology, 1: 1000) for 1 h at room temperature and then stained with diaminobenzidine and hematoxylin.

### 2.15. Statistical Analysis

The GraphPad Prism (version 8.0) software was used for statistical analysis. All in vitro experiments were repeated at least thrice. Variations between two groups were analyzed using two-tailed Student's *t*-test. Analysis of variance was used to compare the differences among multiple groups. Data are shown as the mean ± SD. Statistical significance was set at *P* < 0.05.

## 3. Results

### 3.1. Gypenoside Screening

**Figure 2:**
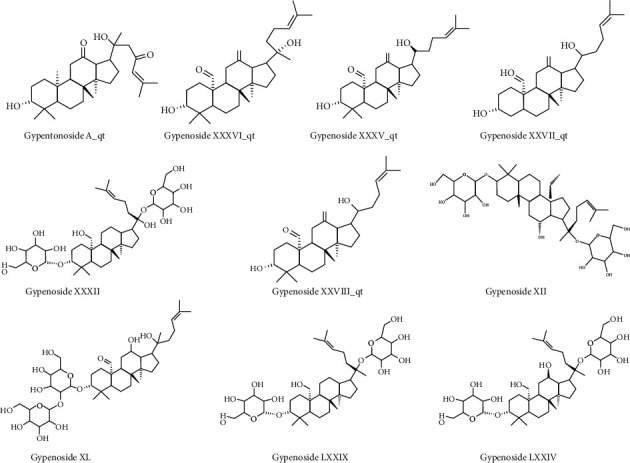
The molecular structures of the 10 selected gypenosides.

As mentioned, OB ≥ 30% and DL ≥ 0.18 were set as the screening conditions. The chemical constituents of the gypenosides were acquired by literature review and database comparison, and 10 ingredients were acquired by preliminary screening (Table 1). The molecular structures of the 10 gypenosides were determined using ChemDraw (Figure 2).

### 3.2. Identification of Gene Targets of Both Gypenosides and Bladder Cancer

**Figure 3:**
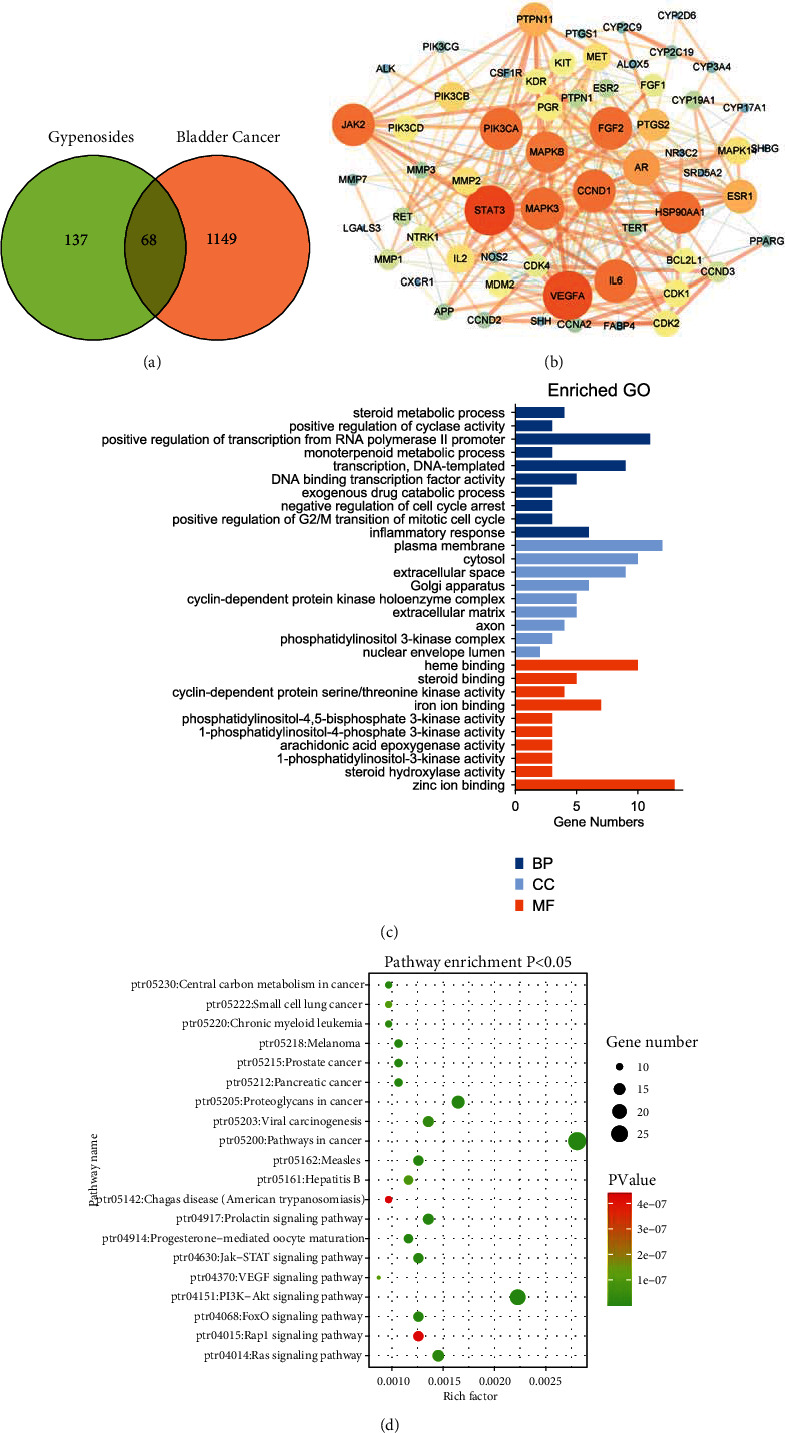
Developing a compound-target network for gypenosides. (a) Venn diagram revealing the overlapping target genes for gypenosides and bladder cancer. (b) PPI network of antibladder cancer-associated proteins. Bigger node sizes and darker red colors indicate a higher degree of association. (c) Gene ontology (GO) functional annotation of potential targets of gypenosides. Cellular components (CC), biological processes (BP), and molecular functions (MF) were analyzed. (d) Kyoto Encyclopedia of Genes and Genomes (KEGG) enrichment analysis for potential signaling pathways of gypenosides. The top 20 pathways with lower *P*-values are visualized.

A total of 205 target genes were identified from the Swiss Target Prediction database based on the 10 identified compounds. We also obtained 8933 bladder cancer-related target genes from the GeneCards database. Duplicate values were deleted when the OMIM and DrugBank databases were combined, and 1217 bladder cancer-related target genes were obtained. A total of 68 potential antibladder cancer target genes were identified by a comprehensive analysis of both the gypenosides and bladder cancer targets (Figure 3(a)).

### 3.3. Construction of a PPI Network of Common Targets

Next, we submitted these genes to the STRING database and constructed a PPI network consisting of 68 nodes and 318 edges (data not shown). We then inputted the above results into the Cytoscape 3.7.1 software to construct and visualize the network. As shown in Figure 3(b), this network contains 10 key nodes: STAT3, VEGFA, PIK3CA, JAK2, CCND1, MAPK3, MAPK8, HSP90AA1, FGF2, and IL6. Thus, we reasoned that these 10 key genes might participate in gypenoside inhibition of bladder cancer.

### 3.4. GO and KEGG Enrichment Analyses

To investigate the specific mechanism through which gypenosides inhibit bladder cancer, we employed DAVID to analyze GO enrichment. We found that 49 terms were associated with BP, nine terms were associated with CC, and 25 terms were associated with MF. The top 10 BPs, MFs, and top nine CCs were ranked based on their *P-values* (Figure 3(c)). Subsequently, KEGG pathway annotation demonstrated that 68 potential target genes were enriched and contributed to 91 pathways. As shown in Figure 3(d), we listed the top 20 pathways based on *P-value*. Analysis of these results revealed that the PI3K-Akt signaling pathway plays a vital role in both gypenoside antitumor activity and bladder cancer survival.

### 3.5. Construction of the Bioactive Compound-Pathway-Target Network

**Figure 4:**
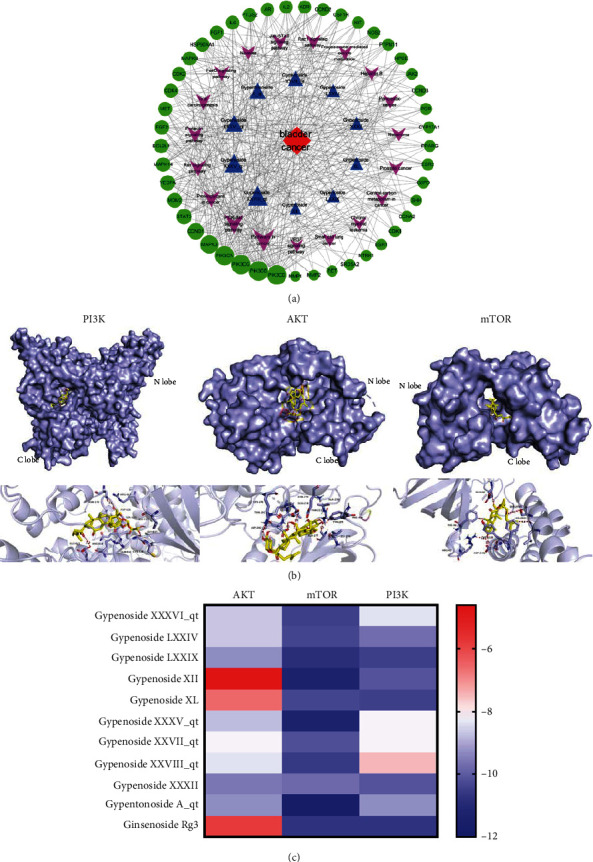
Modeling the potential targets of gypenosides. (a) Network of target genes for gypenosides against bladder cancer. The red diamond represents bladder cancer; blue triangles represent 10 gypenosides; purple arrows represent KEGG pathways; green circles represent the target genes of both gypenosides and bladder cancer. (b) Gypenosides interaction mode with PI3K, AKT, and mTOR, respectively. (c) The binding energy of the components in gypenosides binding with AKT, mTOR, and PI3K.

The compound-pathway-target network was constructed using the Cytoscape 3.7.1 software (Figure 4(a)). Network analysis strongly revealed that Gypenoside XXVIII_qt, Gypenoside XXXV_qt, Gypenoside XXXVI_qt, Gypenoside A_qt, and Gypenoside XXVII_qt were predicted to be the major active ingredients acting against bladder cancer. Importantly, PIK3CA was predicted to be the main target, and MAPK3, CCND1, STAT3, MDM2, and VEGFA were also identified as relatively important targets. The contents of the active components are listed in Table S1.

### 3.6. Molecular Docking Verification

The PI3K/AKT/mTOR axis is an important intracellular signaling pathway that regulates the progression of various cancers [31]. To determine whether gypenosides affect the PI3K/AKT/mTOR pathway, we assessed the binding ability of PI3K, AKT, and mTOR with 10 gypenoside compounds and used ginsenoside Rg3 as a positive control drug (Table S2–S3). The active ingredient with the strongest binding energy for each target is shown in Figure 4(b). The cluster analysis is shown in Figure 4(c). Notably, PI3K, AKT, and mTOR all displayed a strong affinity for gypenosides, indicating that the PI3K/AKT/mTOR pathway is a key point in the gypenoside anticancer process.

### 3.7. Gypenosides Suppress the Proliferation of Bladder Cancer Cells

**Figure 5:**
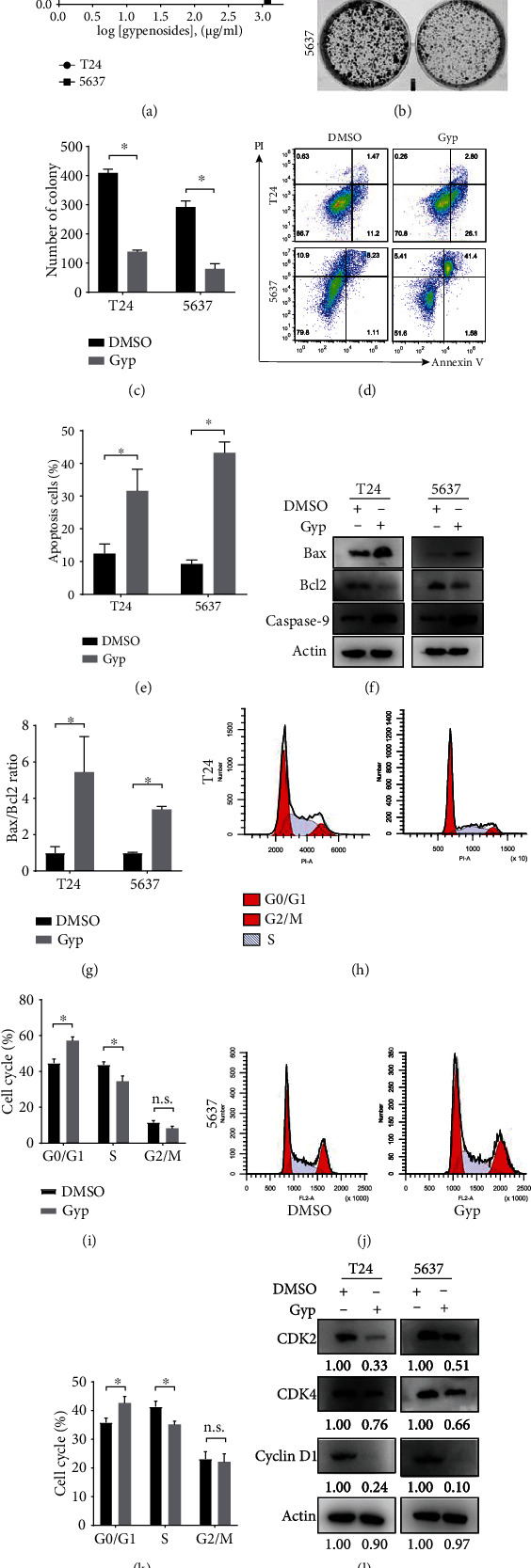
Gypenosides inhibited the proliferation and induced apoptosis of bladder cancer cells. (a) Cells were treated with gypenosides for 48 h, and a CCK8 assay was employed to assess viability. Errors bars show the standard deviation (SD) of the mean of three independent tests. (b) Five hundred cells were plated in a 6-well plate for eight days and treated with gypenosides or DMSO. (c) Quantification of colony formation assays. Student's *t*-test, Error bars show the SD of the mean for three replicate wells, ^∗^*P* < 0.05. (d) Flow cytometry was employed to analyze the staining of propidium iodide (PI) and annexin V in control or gypenoside-treated cells. (e) Quantification of the apoptosis ratio. Student's *t*-test, mean ± SD are given, ^∗^*P* < 0.05. (f) Western blot experiments indicating the expression levels of Bax, Bcl2, and Caspase 9 in the cells treated with DMSO or gypenosides. (g) The Bax/Bcl2 ratio was quantified by ImageJ. Student's *t*-test, mean ± SD are given, ^∗^*P* < 0.05. (h and i) The relative proportion of cell cycle phases in T24 cells treated with DMSO or gypenosides. Mean ± SD are given, n.s., no significant variation, ^∗^*P* < 0.05. (j and k) The relative proportion of cell cycle phases in 5637 cells treated with DMSO or gypenosides. Mean ± SD are given, n.s., no significant variation, ^∗^*P* < 0.05. (l) Immunoblot analysis of the expression of CDK2, CDK4, and Cyclin D1 quantified by ImageJ.

Given that gypenosides are likely to affect the PI3K/AKT/mTOR pathway in bladder cancer cells, which is crucial for cell survival, we evaluated the potential cytotoxic effect of gypenosides in human bladder cancer cell lines. As expected, in the CCK8 assay, gypenosides inhibited the growth of T24 and 5637 cells in a concentration-dependent manner (Figure 5(a)). In clone construction assays, the clone number was much lower in the gypenoside-treated bladder cancer cells than in the control DMSO-treated cells (Figures 5(b) and 5(c)). These results indicate that gypenosides suppress bladder cancer cell growth and proliferation.

### 3.8. Gypenosides Induce Apoptosis and Block the Cell Cycle in Bladder Cancer Cells

Previous studies have shown that gypenosides cause apoptosis in human non-small-cell lung cancer cells and oral cancer cells [1, 32]. To further determine whether gypenosides induce apoptosis in T24 and 5637 cells, we measured apoptosis levels in these cells after gypenoside treatment using the flow cytometry. Notably, the results showed that gypenosides induced a higher rate of apoptosis in bladder cancer cells than DMSO treatment (Figures 5(d) and 5(e)). We also examined protein levels of the apoptotic markers Bcl2, Bax, and Caspase 9. Western blotting indicated that the expressions both of Bax and Casepase 9 were robustly elevated, whereas that of Bcl2 was decreased in gypenoside-treated bladder cancer cells compared to that in the control groups (Figures 5(f) and 5(g)). Moreover, gypenoside-treated cells were more likely to be blocked at the G0/G1 phase of the cell cycle than untreated cells (Figures 5(h)–5(k)). In agreement with its cell cycle-blocking effect, gypenoside treatment significantly reduced the expression of CDK2, CDK4, and Cyclin D1, which are all involved in the G0/G1 cell cycle control (Figure 5(l)). Collectively, these results clearly suggest that in bladder cancer cells, gypenosides induce apoptosis and arrest the cell cycle in the G0/G1 phase.

### 3.9. Gypenosides Inhibit the PI3K/AKT/mTOR Pathway in Bladder Cancer Cells

**Figure 6:**
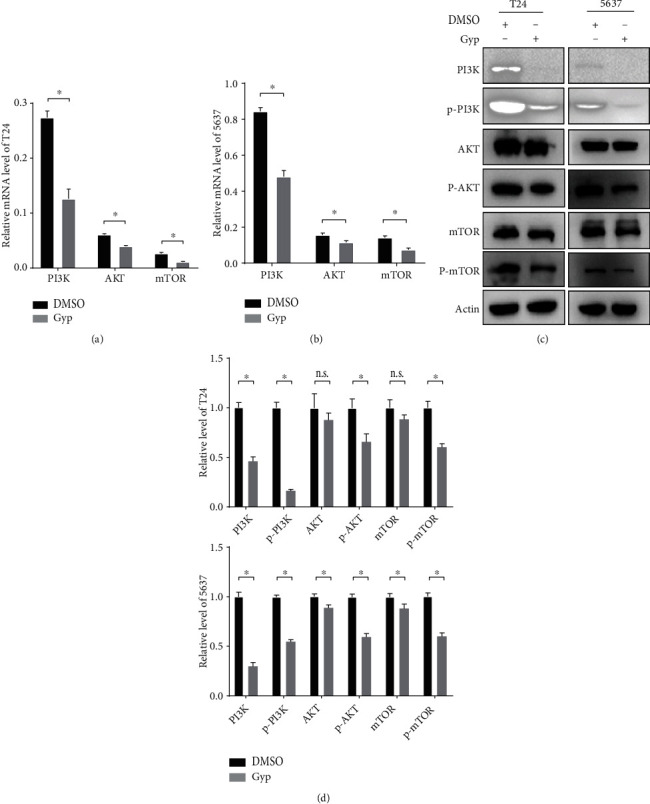
Gypenosides suppress the PI3K/AKT/mTOR pathway. (a and b) RT-qPCR analysis of T24 and 5637 cells expressing the PI3K, AKT, and mTOR. Cells were pretreated with DMSO or gypenosides. Student's *t*-test, mean ± SD are given, ^∗^*P* < 0.05. (c) Immunoblot assay displaying the expression of proteins in 5637 and T24 cells. (d) Quantification of protein expression level. Two-way ANOVA. Error bars, mean ± SD. ^∗^*P* < 0.05.

Next, we assessed whether gypenosides act as tumor suppressors of bladder cancer by repressing PI3K/AKT/mTOR signaling, as predicted by network pharmacology. Indeed, RT-qPCR analysis revealed that gypenoside treatment significantly reduced the mRNA expression of PI3K, AKT, and mTOR in both T24 and 5637 cells compared with that in DMSO-treated cells (Figures 6(a) and 6(b)). Western blot analysis indicated that the expression levels of PI3K, p-PI3K (Y607), p-AKT (Ser473), and p-mTOR (Ser2448) were greatly decreased by gypenoside treatment (Figures 6(c) and 6(d)). These results suggest that gypenosides induce apoptosis in bladder cancer cells by inactivating PI3K/AKT/mTOR signaling.

### 3.10. Gypenosides Inhibit Tumor Growth In Vivo

**Figure 7:**
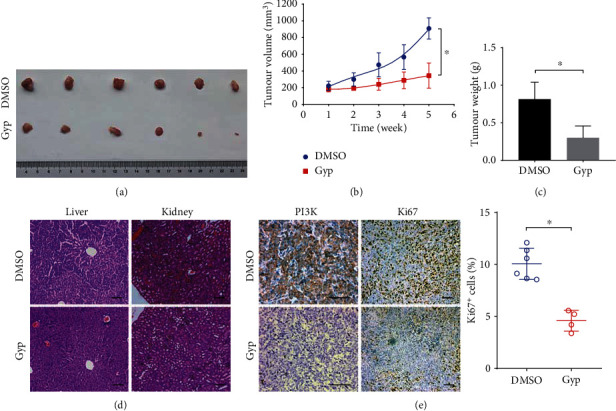
Gypenosides suppresses bladder cancer progression in vivo. (a) The excised and photographed tumors of mice xenotransplanted with human 5637 cells after 21 days of treatment. (b) The tumor volume and weight (c) at the endpoints of subcutaneous xenograft tumors generated by drug treatment and control in nude mice (*n* = 6 for each group). Student's *t*-test, mean ± SD are given, ^∗^*P* < 0.05. (d) Representative images of the HE staining of mice liver and kidney tissues of the different groups. Scale bars, 100 *μ*m. (e) Immunohistochemical staining showing the expression levels of PI3K and Ki-67 in xenograft tumors (*n* = 6 for each group). Scale bars: 100 *μ*m. Student's *t*-test. Error bars, mean ± SD. ^∗^*P* < 0.05.

We further investigated the therapeutic efficacy of gypenosides in athymic nude mice bearing xenograft tumors. Tumor cells were inoculated into the flanks of nude mice and tumor growth was monitored weekly. When the tumors grew to a detectable size, the animals were randomly divided into the control and gypenoside treatment groups. Notably, tumor growth in gypenoside-treated animals was significantly slower in vivo than in the control group (Figures 7(a)–7(c)). At the histological level, gypenosides were not found to cause significant liver or kidney toxicity (Figure 7(d)). Immunohistochemical analysis (IHC) indicated that PI3K was downregulated in the gypenoside-treated group compared to that in the control group (Figure 7(e)). Additionally, cell proliferation was evaluated with Ki-67 staining; and gypenosides greatly reduced the expression of Ki-67 in the subcutaneous tumor tissue (Figure 7(e)). Collectively, these results showed that gypenosides suppressed bladder cancer progression in vivo and exhibited low toxicity.

## 4. Discussion

Network pharmacology, as a system-level polypharmacology approach, is widely applied to identify new therapeutic targets in various complex diseases [33]. In this study, we applied network pharmacology, molecular docking, and biological experiments to determine the active ingredients and molecular mechanisms of gypenosides in bladder cancer. Using network pharmacology, we found that gypenosides may affect PI3K/AKT/mTOR signaling, which is a crucial regulator of bladder cancer cell growth and survival [34]. Next, we verified that gypenosides induced apoptosis and cell cycle blocking in T24 and 5637 bladder cancer cells and caused significant tumor eradication in vivo. Importantly, this finding is consistent with the network pharmacological analysis. Consequently, these findings suggest that gypenosides have a potential therapeutic effect on bladder cancer, and that network pharmacology has credible predictive utility.

Previous studies have indicated that *G. pentaphyllum* can be separated into more than 230 compounds, most of which are saponins, also known as gypenosides [11]. Numerous pure *G. pentaphyllum* compounds have been found to exhibit inhibitory activity against cancer cells in vitro and in vivo [35]. For example, gypenoside L greatly increases the level of intracellular reactive oxygen species (ROS), which, in turn, induces ubiquitination of target proteins, triggers endoplasmic reticulum release of Ca^2+^, and finally results in cell death [36]. Lin et al. revealed that gypenoside increases Bax levels, decreases Bcl2 levels, and induces apoptosis in human myeloid leukemia cells [37]. In addition, gypenosides were shown to increase sensitivity to 5-fluorouracil to stop colorectal cancer cell proliferation in vitro and in vivo [38]. Thus, the antitumor effects of gypenosides have been observed in various types of cancers.

The PI3K/AKT/mTOR pathway is a crucial regulator of multiple cellular processes, including motility, growth, metabolism, and angiogenesis [39, 40]. In bladder cancer, PI3K/AKT/mTOR signaling was observed to be constitutively activated in more than 40% of cases [41]. For instance, PIK3CA encodes the p110*α* subunit of PI3K, whose mutations are found in 21–25% of patients with muscle-invasive bladder cancer [42]. It is now widely accepted that continued smoking is a risk factor for initiating bladder cancer, and Kazuyuki et al. corroborated that nicotine activation of the PI3K/AKT/mTOR signaling pathway in human bladder cancer resulted in increased cancer cell vitality and induced acquired chemoresistance [43]. Based on these findings, a mechanistic understanding of the PI3K signaling pathway in bladder cancer may accelerate the development of new therapeutic strategies.

Previous studies have indicated that the PI3K/AKT/mTOR pathway is one of the most important pathways in cancer progression, while it is also one of the most promising targets for cancer therapy [44]. Ross et al. evaluated the role of the PI3K inhibitor (GDC0941) in the treatment of bladder cancer and showed that bladder cancer cells with activated PIK3CA mutations were sensitive to PI3K inhibitors [45]. Temsirolimus, an inhibitor of mTOR, was previously found to benefit patients with bladder cancer who were resistant to platinum-based chemotherapy [46]. Moreover, earlier studies revealed that the bladder cancer cell line TCCSUP, containing the PIK3CA E545K mutation, was more sensitive to the small-molecule inhibitor pictilisib than wild-type cells in a patient-derived xenograft mouse bladder cancer model [47]. Therefore, understanding the potential role of the PI3K pathway is essential for the treatment of bladder cancer. Here, we demonstrated that gypenosides may inhibit bladder cancer cell proliferation by inhibiting the PI3K/AKT/mTOR pathway. Our findings indicated that gypenosides may be a potential therapeutic target for bladder cancer treatments.

Our study had several limitations. The mechanism by which gypenosides inhibit PI3K mRNA transcription remains unclear. Further, given the genetic complexity of bladder cancer, other pathways associated with the PI3K/AKT/mTOR pathway may also play a role in the antibladder cancer effect of gypenosides. Moreover, further preclinical studies are needed to understand the clinical application of gypenosides in the treatment of bladder cancer.

## 5. Conclusions

In conclusion, using network pharmacology prediction, molecular docking, and in vitro and in vivo experiments, this study provides a mechanistic interpretation of the increased apoptosis observed in bladder cancer cells treated with gypenosides. We concluded that the PI3K/AKT/mTOR signaling pathway might exert a significant effect on gypenoside-mediated antitumor effects in bladder cancer cells. Therefore, gypenosides may be an attractive avenue for developing effective treatments for bladder cancer.

## Figures and Tables

**Table 1:** Bioactive compounds of gypenosides.

Mol ID	Molecule Name	OB (%)	DL
MOL009888	Gypenoside XXXVI_qt	37.85	0.78
MOL009928	Gypenoside LXXIV	34.21	0.24
MOL009929	Gypenoside LXXIX	37.75	0.25
MOL009938	Gypenoside XII	36.43	0.25
MOL009943	Gypenoside XL	30.89	0.21
MOL009969	Gypenoside XXXV_qt	37.73	0.78
MOL009971	Gypenoside XXVII_qt	30.21	0.74
MOL009973	Gypenoside XXVIII_qt	32.08	0.74
MOL009976	Gypenoside XXXII	34.24	0.25
MOL009986	Gypentonoside A_qt	36.13	0.8

Abbreviations: OB, oral bioavailability; DL, drug-likeness.

## Data Availability

All data generated or analyzed during this study are available from the corresponding authors.

## Supplementary Materials

Supplementary 1 Table S1: degree of 10 active components analyzed by Cytoscape.

Supplementary 2 Table S2: binding energy of ten active gradients and positive control drugs.

Supplementary 3 Table S3: docking parameters in molecular docking.
