# Supplementary material for: Gypenoside-Induced Apoptosis via the PI3K/AKT/mTOR Signaling Pathway in Bladder Cancer
Source: Biomed Res Int. 2022 Mar 29;2022:9304552. doi: 10.1155/2022/9304552 (PMC8984741; doi:10.1155/2022/9304552)
Supplement: Supplementary 1 — Table S1: degree of 10 active components analyzed by Cytoscape. [file 9304552.f1.docx]

Table S1: Degree of 10 active components analyzed by Cytoscape.

| Sorts | Components | Degree |
| --- | --- | --- |
| Saponin | Gypenoside XXVIII_qt | 24 |
| Saponin | Gypenoside XXXV_qt | 22 |
| Saponin | Gypenoside XXXVI_qt | 17 |
| Saponin | Gypentonoside A_qt | 15 |
| Saponin | Gypenoside XXVII_qt | 14 |
| Saponin | Gypenoside LXXIV | 9 |
| Saponin | Gypenoside XXXII | 9 |
| Saponin | Gypenoside XL | 9 |
| Saponin | Gypenoside LXXIX | 8 |
| Saponin | Gypenoside XII | 7 |
