# Supplementary material for: Gypenoside-Induced Apoptosis via the PI3K/AKT/mTOR Signaling Pathway in Bladder Cancer
Source: Biomed Res Int. 2022 Mar 29;2022:9304552. doi: 10.1155/2022/9304552 (PMC8984741; doi:10.1155/2022/9304552)
Supplement: Supplementary 2 — Table S2: binding energy of ten active gradients and positive control drugs. [file 9304552.f2.docx]

Table S2: Binding energy of ten active gradients and positive control drugs.

| Active gradient targets | PI3K (2rd0) | AKT (4GV1) | mTOR (1fap) |
| --- | --- | --- | --- |
| Gypenoside XXXVI_qt | -8.3 | -8.5 | -9.9 |
| Gypenoside LXXIV | -9.3 | -8.5 | -9.8 |
| Gypenoside LXXIX | -9.9 | -9 | -10.2 |
| Gypenoside XII | -9.6 | -5.2 | -10.6 |
| Gypenoside XL | -9.9 | -6.3 | -9.8 |
| Gypenoside XXXV_qt | -8.2 | -8.6 | -10.6 |
| Gypenoside XXVII_qt | -8.2 | -8.2 | -9.7 |
| Gypenoside XXVIII_qt | -7.6 | -8.3 | -10 |
| Gypenoside XXXII | -9.6 | -9.2 | -9.4 |
| Gypentonoside A_qt | -9 | -9 | -11 |
| Ginsenoside Rg3 | -10.1 | -5.8 | -10.1 |
