# Supplementary material for: Gypenoside-Induced Apoptosis via the PI3K/AKT/mTOR Signaling Pathway in Bladder Cancer
Source: Biomed Res Int. 2022 Mar 29;2022:9304552. doi: 10.1155/2022/9304552 (PMC8984741; doi:10.1155/2022/9304552)
Supplement: Supplementary 3 — Table S3: docking parameters in molecular docking. [file 9304552.f3.docx]

Table S3: Docking parameters in molecular docking.

| Targets | Receptor | Grid center | Npts | Spacing |
| --- | --- | --- | --- | --- |
| AKT | 4GV1 | center_x = -20.286 center_y = 3.748  center_z = 11.736 | size_x = 15.00 size_y = 15.00  size_z = 15.00 | 0.375 |
| mTOR | 1fap | center_x =-8.57 center_y =26.90 center_z =36.87 | size_x =22.50 size_y =22.50 size_z =22.50 | 0.375 |
| PI3K | 2rd0 | center_x =67.21 center_y =63.78 center_z =93.71 | size_x =56.25 size_y =56.25 size_z =56.25 | 0.375 |

Abbreviations: Npts, Non-parametric time series.
